# Influencing factors of kinesiophobia in older patients with chronic heart failure: A structural equation model

**DOI:** 10.1002/clc.24024

**Published:** 2023-04-28

**Authors:** Jingwen Qin, Juanjuan Xiong, Chen Chen, Xue Wang, Ya Gao, Yan Zhou, Guixiang Zheng, Kaizheng Gong

**Affiliations:** ^1^ Department of Cardiology Affiliated Hospital of Yangzhou University, Yangzhou University Yangzhou Jiangsu China; ^2^ School of Nursing Yangzhou University Yangzhou Jiangsu China; ^3^ Department of Cardiac surgery Nanjing Drum Hospital, The Affiliated Hospital of Nanjing University Medical School Nanjing Jiangsu China

**Keywords:** aged, chronic heart failure, fear of movement, kinesiophobia, structural equation mode

## Abstract

**Background:**

Our recent study has demonstrated that kinesiophobia is common in Chinese inpatients with chronic heart failure (CHF). Symptoms of heart failure (HF), coping mode, self‐efficacy for exercise (SEE), and social support have been reported to be associated with kinesiophobia. However, little is known about the relationships between these four variables and kinesiophobia in older patients with CHF.

**Objective:**

To test a model of influencing factors of kinesiophobia in older CHF patients.

**Methods:**

A cross‐sectional design was conducted from January 2021 to October 2021. The general information questionnaire, the Chinese version of the Tampa Scale for Kinesiophobia Heart (TSK‐SV Heart‐C), Symptom Status Questionnaire‐Heart Failure, SEE, the Medical Coping Modes Questionnaire, and Social Support Rating Scale were used. Spearman correlation analysis and structural equation model (SEM) were performed for data analysis.

**Results:**

A total of 270 older patients with CHF were recruited. Symptom status of HF (*r* = 0.455, *p* < .01), avoidance coping mode (*r* = 0.393, *p* <.01), and yielding coping mode (*r* = 0.439, *p* < .01) were positively correlated with kinesiophobia, while SEE (*r* = −0.530, *p* < .01), facing coping mode (*r* = −0.479, *p* < .01), and social support (*r* = −0.464, *p* < .01) were negatively correlated with kinesiophobia. SEM analysis showed that social support could affect kinesiophobia through the mediating variables of symptom status of HF, avoidance coping mode, and exercise self‐efficacy.

**Conclusions:**

Symptoms of HF, coping mode, SEE, and social support may play role in kinesiophobia in older CHF patients. We should pay more attention to the synergies among these four variables in the improvement of kinesiophobia.

## Graphical abstract

Psychological stress system model in patients with heart failure.

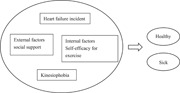

## 1 INTRODUCTION

Currently, more than 64 million people in the world suffer from heart failure (HF),^1^ and the number in China increased to 8.9 million in 2021.^2^ Although the mortality rate of hospitalized HF patients has decreased significantly in recent years,^3^ the demand for cardiac rehabilitation (CR) is increasing. Exercise rehabilitation (ER) is the core component of CR. An accumulating amount of evidence has shown that an increase of 4 MET h/d of activity can reduce the risk of cardiovascular disease death by 12%.^4^ However, the current situation of ER participation is not optimistic. Studies have shown that only 8% of medical units carried out phase I CR and only 4% of the cardiology inpatients participated in CR; furthermore, CR adherence was also not very good.^5^, ^6^, ^7^

Kinesiophobia is an excessive and irrational fear of physical activity or exercises due to the fear of harm or reinjury.^8^ Our recent study demonstrated that the incidence of kinesiophobia was as high as 63.14% in patients with chronic heart failure (CHF).^9^ Kinesiophobia can significantly reduce the participation rate of ER and thus is not conducive to the formation of exercise behavior in patients with heart disease.^10^, ^11^ However, little is known about kinesiophobia among CHF patients; thus, there is insufficient guidance for the formulation of relevant intervention measures.

It is well known that pathological changes in the heart lead to a series of distressful experiences among patients with CHF, such as dyspnea, fatigue, and lower limb edema. Our study showed that fatigue could independently explain 9% of the total variation of kinesiophobia in patients with CHF^9^ and that the severity of fatigue could also indirectly affect patients' physical activity through the mediating effect of kinesiophobia.^12^ However, symptoms of HF include many aspects; therefore, the relationship between symptoms and kinesiophobia still needs further research.

Coping mode serves as an important mediator of stress events and psychosomatic responses. Previous studies have shown that negative coping modes can positively predict the severity of patients' fear ofdisease progression,^13^ while positive coping strategies are significantly negatively correlated with kinesiophobia,^14^ and they directly or indirectly influence the quality of life in patients with breast disease. However, very few studies have explored the impact of coping mode on kinesiophobia among individuals with heart disease.

Self‐efficacy for exercise (SEE) reflects patients' confidence in participating in the exercise in different situations. Patients with higher exercise self‐efficacy are more active regardless of exercise intensity.^15^ Although an improvement in exercise self‐efficacy and a reduction of kinesiophobia were observed among individuals who perform walking exercises based on timing theory,^16^ the relationship between these two parameters remains unclear in patients with CHF.

Social support systems can provide patients with multiple resources and prevent many health problems.^17^, ^18^, ^19^ With adequate social support, adverse emotions can be effectively relieved, thereby decreasing patients' psychological burden.^20^ Among patients with acute heart disease, it has been shown that a higher level of perceived social support is associated with a lower level of kinesiophobia.^21^ However, the mechanism of the effect of social support on kinesiophobia among patients with CHF remains unclear.

Interestingly, the interaction path between the above‐mentioned potential influencing factors and kinesiophobia in older patients with CHF has yet to be fully elucidated. Therefore, the present study aims to explore the mechanism of the relationships between symptoms of HF, SEE, coping mode, social support, and kinesiophobia by performing a structural equation model (SEM). The findings of the current study will provide theoretical guidance for future intervention strategies for kinesiophobia among older CHF patients.

## 2 DESIGN AND METHODS

### 2.1 Theoretical basis and hypothesized model

Based on Lazarus' stress and coping theory, Jiang developed the psychological stress system model (Supporting Information: Figure 1), which shows that internal factors, external factors, life events, stress response, and other factors in the stress system influence and restrict each other.^22^ If the system is unbalanced, it will have a negative impact on the individual's physical and mental health. In this study, symptoms of HF were considered as stressors, kinesiophobia was considered as the negative mental effects of the disease on patients, and SEE was considered as the cognitive evaluation of patients' exercise behavior. Social support and coping modes were also included to explore their influence on kinesiophobia.

Social support buffer theory refers to the notion that when individuals are faced with stressful events, social support can alleviate the negative effects by affecting their cognition or behavior and play a certain role as a stress buffer.^23^ Based on the results of previous literature research, we hypothesized that social support might influence the level of kinesiophobia by regulating the severity of HF symptoms, exercising self‐efficacy, and coping mode. The hypothesized model is shown in Supporting Information: Figure 2.

### 2.2 Study design and objective

This was a cross‐sectional survey. We aimed to examine the relationships among symptom status of HF, SEE, coping modes, social support, and kinesiophobia in older patients with CHF and explored the interaction mechanism among these variables. The protocol was reviewed by the Ethics Committee of the School of Nursing, Yangzhou University. (ethical code: YZUHL2020016).

### 2.3 Participants

A convenience sampling method was used to select older CHF patients hospitalized in the Department of Cardiology of a territory A hospital in Yangzhou, Jiangsu, China, from January to October 2021. Before the study survey began, we explained the purpose of the study to the subjects and informed them that their participation was voluntary. Each participant was required to provide written informed consent. The inclusion criteria were as follows: (I) met the diagnostic criteria of relevant guideline^24^; (II) age 60 years or older; (III) New York Heart Association (NYHA) functional grade II or III; and (IV) conscious and able to complete the questionnaires independently or with the assistance of our researchers. The exclusion criteria were as follows: (I) unable to complete the questionnaires; (II) complicated with other systemic serious diseases or malignant tumors; (III) complicated with musculoskeletal diseases that seriously affect their mobility; and (IV) declined to participate.

When conducting SEM, it is recommended to obtain at least 200 samples to obtain stable model results, and 5–20 times the total number of variables is also recommended in the process of sample size determination.^25^ In our study, there were a total of 21 parameters and 10 observations for each parameter, thus indicating that 210 patients were needed. Considering a 20% rate of invalid data, 252 was the minimum sample size. We ultimately collected 270 valid questionnaires.

### 2.4 Measurements

#### 2.4.1 The demographic and clinical questionnaire

The demographic and clinical questionnaires were designed by the current research group. The demographic information included age, gender, educational background, occupation before retirement, body mass index, and monthly family income. The clinical data included the number of readmissions over the past year, disease course, and NYHA classification.

#### 2.4.2 Chinese version of the Tampa Scale for Kinesiophobia Heart (TSK‐SV Heart‐C)

The Tampa Scale for Kinesiophobia Heart (TSK‐SV Heart) was mainly used to assess exercise fear in patients with heart disease. It was originally developed by Bäck et al. for coronary heart disease in 2012 and translated into Chinese in 2019.^11^, ^26^ The TSK‐SV Heart‐C includes four dimensions, that is, danger perception, motion avoidance, fear of injury, and functional disorder; the cutoff point is 37, and it has been tested for various heart diseases in China.^16^, ^27^ Higher scores indicate a more severe level of kinesiophobia. In this study, Cronbach's *α* was .899.

#### 2.4.3 Symptom Status Questionnaire‐Heart Failure (SSQ‐HF)

SSQ‐HF was first designed by Heo et al. in 2015.^28^ This questionnaire assesses the seven most common symptoms in patients with HF: shortness of breath during the daytime, shortness of breath when lying down, fatigue or lack of energy, chest pain, leg or ankle swelling, difficulty sleeping at night, and dizziness or loss of balance. This questionnaire can measure HF patients' symptoms for the last 4 weeks across three aspects: frequency, severity, and the degree of distress brought to patients with HF symptoms. The score for each symptom was 0–12, and the total score of the questionnaire was 0–84. In this study, Cronbach's *α* was .785.

#### 2.4.4 Medical Coping Modes Questionnaire (MCMQ)

The MCMQ was first established in 1987 and revised by Jiang's team in the Chinese context in 2000.^29^, ^30^ It is mainly used to evaluate patients' coping strategies in different situations. This scale consists of 20 items across 3 dimensions: facing, avoiding, and yielding. A 4‐point Likert scale was used for each item and a higher score on a specific dimension means that the patient tends to choose that coping strategy. The MCMQ has been widely used in China and the Cronbach's *α* for the three dimensions in this study were .762, .740, and .861, respectively.

#### 2.4.5 Self‐efficacy for Exercise (SEE)

The SEE scale was specifically developed for older people by Resnick and Jenkins.^31^ It contains nine items and each item scores from 0 to 10 points, representing patients' exercise confidence in different situations, ranging from “little confidence” to “very confidence.” The final score is based on the average score of nine items. Higher scores indicate a higher level of self‐efficacy. The SEE scale was translated into Chinese by a Taiwanese scholar in 2009.^32^ The Cronbach's *α* was .874 in the present study.

#### 2.4.6 Social Support Rating Scale (SSRS)

The SSRS was first developed by Professor Xiao.^33^ This scale contains 10 items and across 3 dimensions, namely objective social support, subjective social support, and the utilization of social support. The total score on the scale ranges from 11 to 72, and a higher score indicates a higher level of social support. The Cronbach's *α* of the SSRS in this study was .709.

### 2.5 Data collection

Questionnaires were distributed to the patients through face‐to‐face interviews. Any questions were explained in detail before the investigation began. The researchers did not give any suggestive guidance during the filling process. Questionnaires were completed with the help of our investigators if patients encountered difficulties during the process and they were reminded if there were any missing items. Clinical data were obtained from electronic medical records by two researchers.

### 2.6 Statistical analysis

IBM SPSS (version 26.0) software was used for data analysis. Descriptive analysis was used for patients' demographic and clinical characteristics. Spearman correlation analysis was conducted to analyze the correlation relationships among the five scales. SEM was conducted with AMOS24.0 software. The normed *χ* ^2^ (*χ* ^2^/*df*), root mean square error of approximation (RMSEA), goodness‐of‐fit index (GFI), normed fit index (NFI), and comparative fit index (CFI) were selected to evaluate the model fit degree. Their values should meet the following standards: *χ* ^2^/*df* < 3, RMSEA < 0.08, and GFI, NFI, and CFI values >0.8 indicate that the model fit is acceptable and >0.9 indicate excellent. The mediation effect was examined using the bootstrap approach.

## 3 RESULTS

### 3.1 Sample characteristics

In this study, a total of 290 questionnaires were distributed. After excluding 20 invalid questionnaires, 270 questionnaires were eventually included in the data analysis. The average age of the subjects was 76.17 ± 7.96. Most of the subjects were female, and 83.33% had a junior high school education or below. More than half of the patients had a monthly family income ≥3000 yuan RMB, and 23.33% of the patients had been hospitalized for HF more than three times in the past year. Additionally, 32.22% of the patients had a duration of more than 5 years and 82.22% had a cardiac function of NYHA Ⅲ (see Table 1).

**Table 1:** Sociodemographic and disease characteristics of older patients with CHF (*N* = 270).

Variables	Category	*N*	% or mean (SD)
Age, years	–	270	76.17 (7.96)
Gender	Male	131	48.52
	Female	139	51.48
Educational background	Junior high or below	225	83.33
	High school or above	45	16.67
Occupation before retired	Unemployed	20	7.41
	Physical labor	191	70.74
	Mental labor	59	21.85
Body mass index	<18.5	18	6.67
	18.5–23.9	120	44.44
	≥24	132	48.89
Monthly family income, yuan	<3000	110	40.74
	≥3000	160	59.26
Number of readmissions over the past year, times	1–2	207	76.67
	≥3	63	23.33
Duration, years	<5	183	67.78
	≥5	87	32.22
NYHA classification	Ⅱ	48	17.78
	Ⅲ	222	82.22

Abbreviations: CHF, chronic heart failure; NYHA, New York Heart Association.

### 3.2 The correlational association among different variables

As shown in Table 2, the symptom status of HF (*r* = 0.455, *p* < .01), avoidance coping mode (*r* = 0.393, *p* < .01), and yielding coping mode (*r* = 0.439, *p* < .01) were positively correlated with kinesiophobia, while a negative correlation between SEE (*r* = −0.530, *p* < .01), facing coping mode (*r* = −0.479, *p* < .01), social support (*r* = −0.464, *p* < .01), and kinesiophobia was observed.

**Table 2:** Correlational relationships among different variables.

	1	2	3	4	5	6	7
1	1						
2	−0.310**	1					
3	−0.191**	0.282**	1				
4	0.112	−0.113	−0.267**	1			
5	0.235**	−0.301**	−0.245**	0.509**	1		
6	−0.233**	0.318**	0.519**	−0.299**	−0.356**	1	
7	0.455**	−0.530**	−0.479**	0.393**	0.439**	−0.464**	1

*Note*: 1, symptom status of heart failure; 2, self‐efficacy for exercise; 3, facing coping mode; 4, avoiding coping mode; 5, yielding coping mode; 6, social support; 7, kinesiophobia.

** *p* < .01.

### 3.3 Construction of the model of influencing factors of kinesiophobia in older CHF patients

**Figure 1:**
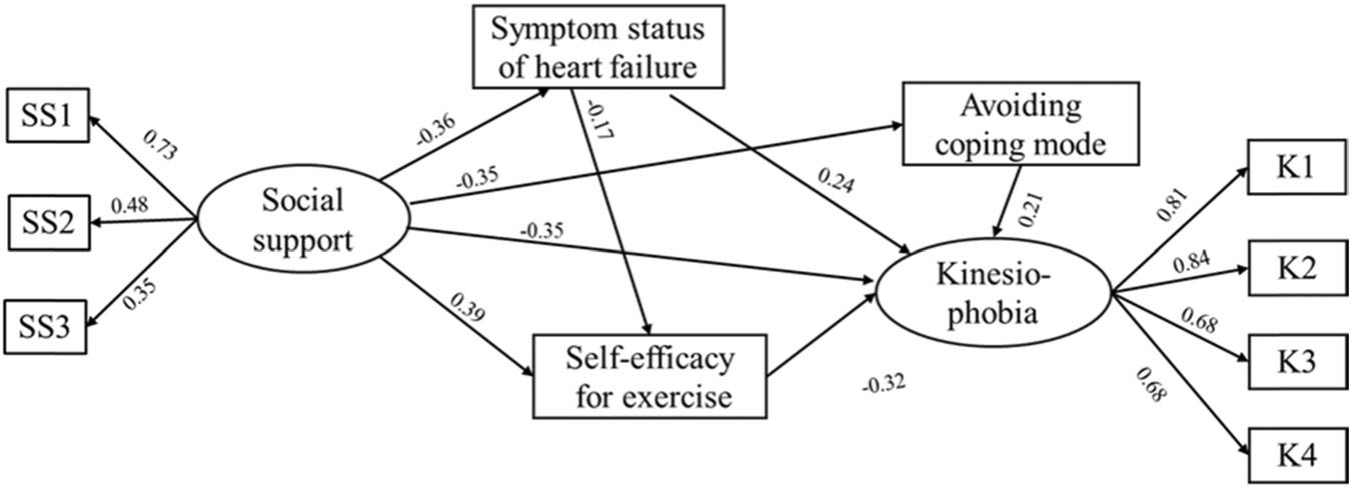
The definitive construction equation model with standardized paths. SS1, subjective social support; SS2, objective social support; SS3, utilization of social support; K1, danger perception; K2, motion avoidance; K3, fear of injury; K4, functional disorder.

The maximum likelihood method was used to evaluate the SEM. The results of the definitive model and the parameter estimates are shown in Figure 1 and Table 3. The fitting degree between the tested data and the hypothesis model was satisfactory (*χ* ^2^/*df* = 2.024, RMSEA = 0.062, GFI = 0.954, NFI = 0.926, CFI = 0.961). Our definitive model showed that social support, symptom status of HF, SEE, and avoidance coping mode could directly influence the level of kinesiophobia (*β* = −.35, .24, −.32, .21, *p* < .01). In addition, social support could indirectly affect kinesiophobia through four different mediation paths (Figure 1).

**Table 3:** Structural parameter estimates of the definitive model.

Hypothesized relationship	*β*	SE	*t* Value	*p* Value
A → B	−.36	1.316	−3.404	<.001
A → C	−.35	0.389	−3.562	<.001
A → D	.39	0.079	3.613	<.001
A → E	−.35	0.241	−3.282	.001
B → D	−.17	0.004	−2.724	.006
B → E	.24	0.010	4.425	<.001
C → E	.21	0.033	3.664	<0.001
D → E	−.32	0.194	−5.125	<0.001

*Note*: A, social support; B, symptom status of heart failure; C, avoiding coping mode; D, self‐efficacy for exercise; E, kinesiophobia.

### 3.4 The examination results of the mediating effects

In AMOS24.0 software, 5000 bootstrap samples were performed and the mediation effect was assessed using bias‐corrected 95% conidence interval (CI). As shown in Table 4, the upper and lower bounds of the 95% CI of the four mediation paths did not contain 0, indicating that the indirect effects were all significant.^34^ Social support has the largest mediating effect on kinesiophobia through exercise self‐efficacy, accounting for 40.9% of the total effect.

**Table 4:** The direct, indirect, and total effects of variables in the definitive model.

Pathways	Standardized direct effects	Standardized indirect effects	Standardized total effects	Bias‐corrected	
				Lower	Upper
A → B	−0.359	–	−0.359		
A → C	−0.352	–	−0.352		
A → D	0.393	0.062	0.455	–	–
A → E	−0.352	−0.308	‐0.660	–	–
B → D	−0.173	–	−0.173		
B → E	0.244	0.055	0.299	0.011	0.110
C → E	0.209	–	0.209		
D → E	−0.319	–	−0.319		
A → B → E	–	−0.088	–	−0.148	−0.050
A → C → E	–	−0.074	–	−0.128	−0.039
A → D → E	–	−0.126	–	−0.199	−0.075
A → B → D → E	–	−0.020	–	−0.043	−0.006

*Note*: A, social support; B, symptom status of heart failure; C, avoiding coping mode; D, self‐efficacy for exercise; E, kinesiophobia.

## 4 DISCUSSION

To the best of our knowledge, the present study was the first to use social support buffering theory to examine the interaction mechanism among various influencing factors of kinesiophobia in older patients with CHF. Our results indicated that social support, symptom status of HF, SEE, and avoidance coping mode were influencing factors of kinesiophobia in older CHF patients, and social support could affect patients' kinesiophobia level through four different mediation paths.

The results of this study showed that the symptoms of HF in older patients with CHF were positively correlated with kinesiophobia. Symptom management has a practical significance for patients' physical and mental health.^35^ However, most CHF patients have a poor understanding, interpretation, and response to various symptoms regardless of their age.^36^ The results of our questionnaire revealed that dyspnea and fatigue were the most frequent and serious symptoms. Due to impaired cardiac function, patients often have obvious respiratory symptoms after activity, such as palpitation, chest tightness, and shortness of breath. These discomfort experiences can seriously hinder their enthusiasm for physical activity.^37^ Fatigue, often accompanied by obvious exercise intolerance,^38^ can also affect patients' judgment of their exercise ability, and exercise avoidance may occur.^39^, ^40^ In addition, we found that social support could affect kinesiophobia by influencing the severity of HF symptoms. Social support is significantly positively correlated with symptom management self‐efficacy.^41^ An effective social support system can improve patients' confidence in disease self‐care and symptom management, which is conducive to reducing the severity of clinical symptoms. In older CHF patients, kinesiophobia was mainly caused by the fear of cardiopulmonary response during exercise.^42^ Therefore, making full use of patients' social support system to assist patients in symptom management may effectively reduce their kinesiophobia.

Our results revealed that coping mode was one of the influencing factors of kinesiophobia. Coping style belongs to the synthesis of human cognition and behavior, and often influences patients' psychology and behavior style as an intermediary variable.^22^ It has been shown that the coping style of facing has a positive adjustment effect on sports psychological fatigue; in contrast, avoiding or yielding coping strategies will increase sports‐related fatigue,^43^ which is consistent with the results of the present study. In addition, we found that social support could indirectly affect kinesiophobia through the coping style of avoidance. A good social support network can enhance patients' sense of security in the psychological state, which can lead to positive coping strategies rather than negative coping strategies. This may be useful in regulating the sense of fatigue in sports psychology and reducing patients' kinesiophobia level.

The present study indicated that exercise self‐efficacy was negatively correlated with kinesiophobia. Exercise self‐efficacy can independently affect the frequency, duration, and intensity of activities when CHF patients are in the family recuperation stage.^44^ CHF patients with higher exercise self‐efficacy had stronger exercise confidence and their level of kinesiophobia may be correspondingly lower under different conditions.^45^ In addition, we found that social support could indirectly affect kinesiophobia through exercise self‐efficacy, which accounted for the highest proportion (40.9%) in the total indirect effect of social support on kinesiophobia. Different aspects of social support can enhance patients' exercise motivation,^46^ and caregivers' participation in exercise can also improve their exercise self‐efficacy.^44^ These benefits from social support may offer the possibility of improving kinesiophobia. Therefore, arousing the social support system may be a practical strategy for improving exercise self‐efficacy and reducing kinesiophobia, which contributes to promoting the transformation from exercise motivation to behavior.^47^

Notably, our results showed that social support could influence kinesiophobia through the chain mediating effect of HF symptoms and exercise self‐efficacy, suggesting that adequate social support can alleviate clinical symptoms by improving their symptom management behavior, and symptom relief may further improve patients' exercise self‐efficacy, enhance exercise confidence, and then reduce kinesiophobia. While guiding patients to obtain and use effective social support, clinical workers can also strengthen health education on symptom management of HF and formulate interventions to improve exercise self‐efficacy. These measurements may play a synergistic role in the whole nursing process and more effectively reduce the level of kinesiophobia in older CHF patients.

## 5 LIMITATIONS

We should be cautious when generalizing the results of this study due to the following limitations. First, a convenience sampling method and a single‐center design were adopted in this study, so the results were not sufficiently representative. Second, this study used a cross‐sectional design, so the causality of each variable in the SEM cannot be determined. Third, some demographic characteristics may have an impact on kinesiophobia, such as educational background and family monthly income, but they were not included in the SEM. Lastly, subjects enrolled in our research were all aged CHF patients who might not consider CR as a means of exercise, so interventions based on these four variables to improve kinesiophobia might be only discussed in regard to daily physical activities.

## 6 CONCLUSIONS

Under the guidance of the psychological stress system model and social support buffer theory, we tested an SEM model of the influencing factors of kinesiophobia in elderly patients with CHF. Our results suggest that a comprehensive management plan should be developed when improving kinesiophobia, including helping patients manage their symptoms well, making full use of social support systems, enhancing their exercise confidence, and guiding them to actively cope with the uncomfortable experience brought by the disease in different ways. Although SEM confirmed that there was a certain relationship between kinesiophobia and the above four variables, the variables may change over time. Therefore, a longitudinal study design should be performed in the future. In addition, the specific impact of kinesiophobia on exercise behavior in older CHF patients also needs to be confirmed in the future.

## CONFLICT OF INTEREST STATEMENT

The authors declare no conflict of interest.

## Supporting information

Supporting information.

## Data Availability

The data that support the findings of this study are available from the corresponding author upon reasonable request.
