# Supplementary material for: Influencing factors of kinesiophobia in older patients with chronic heart failure: A structural equation model
Source: Clin Cardiol. 2023 Apr 28;46(7):729–36. doi: 10.1002/clc.24024 (PMC10352966; doi:10.1002/clc.24024)
Supplement: Supplementary file 1 — Supporting information. [file CLC-46-729-s001.docx]

Stressor life event

External factors

social support

environment

……

Internal factors

cognitive evaluation

coping mode

personality

……

……

Stress response

Healthy

Sick

**Supplementary figure 1 Psychological Stress System Model**

Symptom status of

heart failure

Social support

Coping mode

Self-efficacy for exercise

Kinesiophobia

**Supplementary figure 2 The hypothesized model**
